# Markov models of the *apo*-MDM2 lid region reveal diffuse yet two-state binding dynamics and receptor poses for computational docking

**DOI:** 10.1038/srep31631

**Published:** 2016-08-19

**Authors:** Sudipto Mukherjee, George A. Pantelopulos, Vincent A. Voelz

**Affiliations:** 1 Department of Chemistry, Temple University, Philadelphia, PA, USA.

## Abstract

MDM2 is a negative regulator of p53 activity and an important target for cancer therapeutics. The N-terminal lid region of MDM2 modulates interactions with p53 via competition for its binding cleft, exchanging slowly between docked and undocked conformations in the absence of p53. To better understand these dynamics, we constructed Markov State Models (MSMs) from large collections of unbiased simulation trajectories of *apo*-MDM2, and find strong evidence for diffuse, yet two-state folding and binding of the N-terminal region to the p53 receptor site. The MSM also identifies *holo*-like receptor conformations highly suitable for computational docking, despite initiating trajectories from closed-cleft receptor structures unsuitable for docking. Fixed-anchor docking studies using a test set of high-affinity small molecules and peptides show simulated receptor ensembles achieve docking successes comparable to cross-docking studies using crystal structures of receptors bound by alternative ligands. For p53, the best-scoring receptor structures have the N-terminal region lid region bound in a helical conformation mimicking the bound structure of p53, suggesting lid region association induces receptor conformations suitable for binding. These results suggest that MD + MSM approaches can sample binding-competent receptor conformations suitable for computational peptidomimetic design, and that inclusion of disordered regions may be essential to capturing the correct receptor dynamics.

Under normal cellular conditions, the tumor suppressor protein p53 is kept at a low basal level in part due to downregulation by MDM2 (mouse double minute 2 homolog), an E3 ubiquitin ligase that recruits p53 for degradation via direct interaction with the p53 transactivation domain (TAD). Since many tumor cells still retain wild-type p53, a promising avenue of cancer treatment is to restore p53 activity by blocking the MDM2-p53 interaction with high-affinity MDM2-binding ligands.

A high-resolution x-ray crystal structure of p53 TAD bound to MDM2 in a helical conformation has been available for some time, and has spurred widespread effort towards developing inhibitors that potently disrupt p53-MDM2 binding^1^. In addition to small molecules^2^,^3^. peptidomimetics have been designed to mimic the p53 helix, such as stapled peptides^4^, beta-peptides^5^, spiroligomers^6^, high-affinity D-peptides^7^,^8^,^9^, arylamides, terphenyls, hydrogen-bond surrogates^10^ and oligooxopiperazines^11^, many of which were developed as a result of-or in concert with-computational modeling and design^3^,^11^,^12^,^13^,^14^,^15^,^16^.

Aside from its therapeutic interest, the p53-MDM2 interaction has served as a valuable model system for understanding protein-protein interactions, especially for intrinsically disordered proteins such as the p53 TAD that fold upon binding^17^. Underscoring the importance of this work is recent evidence that residual helicity in the p53 TAD directly alters cell signaling *in vivo*^18^. Similarly, consideration of intrinsic disorder is important to understanding MDM2, as it contains an unstructured N-terminal lid region (residues 1–25) which competes with p53 for the binding cleft. In the absence of p53, quantitative NMR spectroscopy has shown transient structuring and binding of the lid region to the p53 cleft on slow (>10 ms) exchange timescales, consistent with the structuring of a short, well-ordered helix in residues 19–24 (Fig. 1)^19^. Recent NMR and X-ray co-crystal structures have revealed that small-molecule inhibitors can induce structuring of the lid region through specific favorable interactions^20^, suggesting that computational prediction of lid region structure and dynamics could be very useful for computational design.

Here, to better understand the structure and dynamics of the N-terminal lid region of *apo*-MDM2, we perform extensive simulation studies to characterize the mechanism of association with the p53 binding cleft, and explore the possible role of such computational studies in drug discovery. From many independent trajectories of MDM2 starting from the *apo* state obtained by parallel distributed simulation, we construct a Markov State Model (MSM) of N-terminus dynamics that predicts two-state binding to the p53 cleft, in agreement with experimental findings. We then explore the utility of the MSM for *in silico* drug discovery by performing computational docking studies to kinetic metastable states of the MDM2 receptor. Remarkably, our findings suggest that the ensemble of metastable receptor conformations identified in the MSM can be used to achieve docking results similar to or better than cross-docking studies of crystal structures, and moreover, that inclusion of the N-terminus is essential in selecting open-cleft receptor conformations suitable for docking.

## Results

### Markov State Model (MSM) analysis of simulated *apo*-MDM2 dynamics predicts two-state binding of the lid region to the p53 cleft

MSMs describe conformational dynamics as a network of transitions between kinetically metastable states^21^. To construct an MSM of N-terminal dynamics from simulation data, trajectory snapshots are first assigned to metastable conformational states. To identify these metastable states, we first used tICA^22^,^23^ to find a low-dimensional subspace reflecting the slowest conformational motions of the N-terminal region (residues 1–25) and residues in the binding site. Projections to the two largest components (tIC1 and tIC2) were subsequently used for conformational clustering into 2000 metastable microstates, and for visualizing the folding/binding landscape.

Next, observed transitions between states are used to infer a transition matrix **T**^(*τ*)^, whose elements *T*_*ji*_ contain the probability of transitioning from state *i* to state *j* within time *τ*. The right (*ϕ*_*n*_) and left (*ψ*_*n*_) eigenvectors of the transition matrix yield a complete description of state population dynamics, via the chemical master equation, *d***p**/*dt* = **Kp**, where **T** = exp(*τ***K**), whose solution is


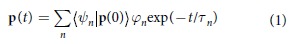


**Figure 2:**
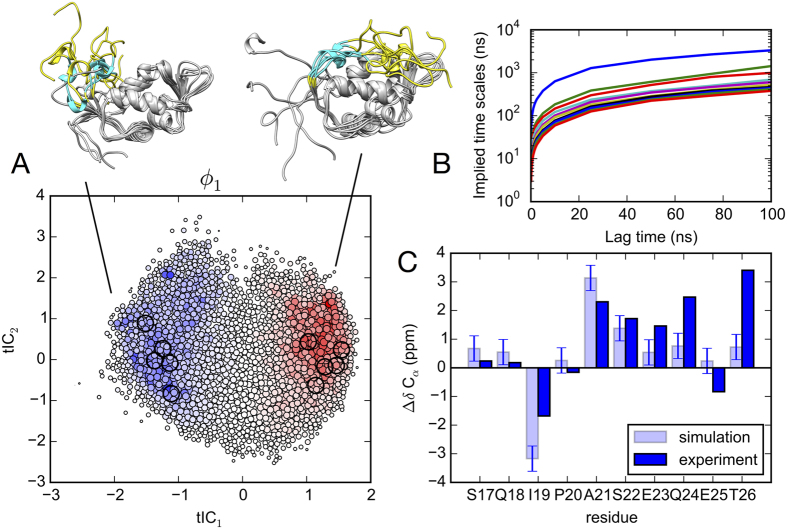
(**A**) Projection of the 2000 MSM microstates (filled circles) to tIC_1_ and tIC_2_ coordinates. The size of each circle is proportional to the equilibrium population, and is colored according to the slowest relaxation eigenmode, *ϕ*_1_. Population flux along this mode is from blue to red, representing a transition from unbound to bound states of the lid region, which we visualize using five representative structures from each basin. (**B**) Implied timescales versus MSM lag time show a clear gap indicating apparent two-state kinetics. (**C**) Simulation predictions of C_*α*_ chemical shift deviations from random coil for the lid region (residues 17–26, cyan ribbon in panel **A**) calculated by SHIFTX2^54^ agree with experimental values^19^.

Here, **p**(0) is a vector of initial state populations at time *t* = 0, and the implied timescales *τ*_*n*_ = −*τ*/1n *μ*_*n*_ associated with each eigenmode *n* are related to the eigenvalues *μ*_*n*_ of T. We define the sign structure of each eigenvector such that 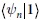 is positive, so that dynamics (starting from a hypothetical uniform distribution) can be described as a superposition of positive-amplitude eigenmodes *ϕ*_*n*_, each decaying at time scale *τ*_*n*_. The stationary eigenvector (i.e. the equilibrium state populations) is *ϕ*_0_, for which *τ*_*n*_ = ∞. The resulting MSM shows a two-state mechanism for the lid region binding to the cleft. The sign structure of the slowest relaxation eigenmode *ϕ*_1_ shows population flux from unbound to bound states of the lid regions, indicated by two diffuse basins aligned with tIC_1_, the degree of freedom representing the slowest conformational motions (Fig. 2a). Interestingly, compared to the tICA landscapes reported for many protein folding systems^24^,^25^,^26^, the lid landscape is remarkably diffuse, even in the secondary eigenmodes (Figures S4 and S5), reflecting the lack of residual structure. Similar landscapes have been found in other MSM studies of disordered proteins^27^,^28^. Implied timescales computed at lag times ranging from 100 ps to 100 ns show a clear gap between the slowest and next-slowest implied timescale, indicative of apparent two-state dynamics (Fig. 2b). The slowest implied timescale is close to 1 *μ*s, comparable to the molecular on-rate of a peptide at high effective concentration. This timescale is over four orders of magnitude faster than the slow (>10 ms) conformational exchange of residues 19–24 reported by Showalter *et al*., which suggests that our simulation trajectories, each shorter than 1 *μ*s, do not capture rare unstructuring events expected in this region. Nevertheless, the simulations show good agreement with experimentally measured chemical shifts in this regions for the *apo* state, which is estimated to have ~90% of the lid population in an associated state.

The next-slowest eigenmode relaxation, *ϕ*_2_, reflects conformational dynamics of the lid region along the tIC_2_ component, and it is similarly diffuse (Figure S4). To gain structural insight into these motions, we performed secondary structure analysis and Bayes Factor analysis^25^ of interresidue contacts formed along different quadrants of the tICA projection (SI Text, Figures S4 and S5). While the slowest relaxation (along tIC1) corresponds to disassociation of the N-terminus from the C-terminus, structuring of the lid region into a helix, and association with the binding cleft helix *α*_2_, the next-slowest relaxation (along tIC_2_) largely reflects an increase in average self-association of the lid region, with an increase in sheet structure.

### Computational docking of known MDM2 ligands to simulated receptor ensembles achieves success comparable to crystal structure cross-docking

Virtual screening studies rely heavily on the availability of high-resolution crystal structures. Since the MDM2 trajectories were initiated from an *apo* NMR structure (PDB: 1Z1M) with a closed binding cleft unsuitable for computational docking, our work presents an excellent opportunity to test how successfully an MD + MSM approach can be used as a refinement procedure to achieve high-quality receptor structures for docking.

To evaluate the quality of simulated receptor structures, we used the DOCK6 algorithm to perform computational docking of a test set of 10 ligands to the 2000 MSM microstate structures (with the lid region removed). Our test set consisted of eight small-molecule ligands and two peptide ligands, all with high-resolution crystal structures (Table 1). The small-molecule ligands include, among others, the best-in-class inhibitor nutlin, and similar compounds. The peptide ligands include the native p53 fragment^29^, and a high-affinity designed inhibitor sequence, PMI N8A^30^. Several modifications were made to standard docking procedures to facilitate the efficient docking of peptide sequences, most notably: fixing backbone atoms in their helical conformation via an artificial cyclization bond between terminal alpha-carbons, while retaining side chain rotamer search (see Methods).

**Figure 3:**
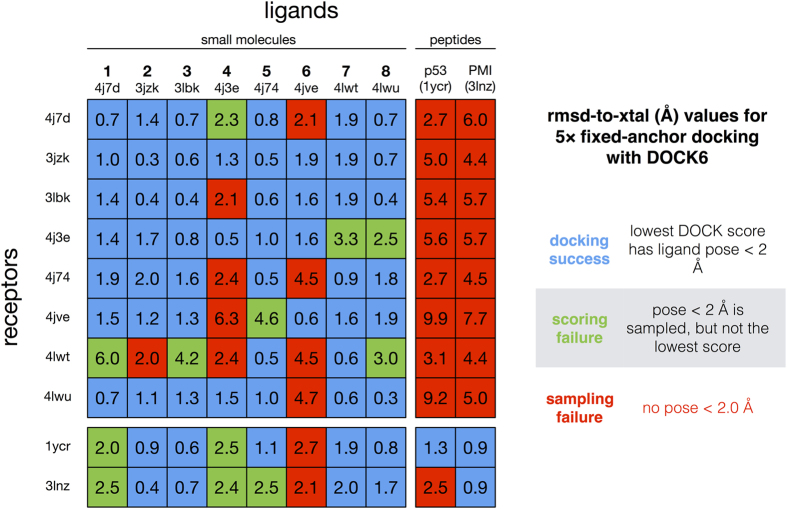
Self-docking and cross-docking results for a test set of 10 MDM2 ligands (Table 1) with available co-crystal structures (listed by PDB ID). Values shown are the rmsd (in Å) of the best-scoring docked ligand pose to the crystal ligand pose. Docking successes are shown in blue, scoring failures are shown in green, and sampling failures are shown in red.

To establish the baseline accuracy of the DOCK algorithm for this system, ligands were re-docked to their own co-crystal structures, and cross-docked to all other receptor structures in the test set (Fig. 3).

In all cases, the best re-docking scores corresponded to a correctly docked pose, which we define as having an rmsd of 2.0 Å or less to the crystal pose, thus validating the accuracy of DOCK. Cross-docking results show the inherent variability of docking to a target receptor structure, and show that some MDM2 receptor crystal structures are more likely to produce false positives or outright failures when non-native ligands are docked. Cross-docking is the least successful for small-molecule docking to peptide-bound receptor structures, and vice versa. We also cross-docked all the ligands in the test set to the *apo*-MDM2 receptor structure (PDB:1Z1M, with the lid region removed), which confirmed its unsuitability for docking; best-scoring poses for all ligands showed rmsd values >5.4 Å.

**Figure 4:**
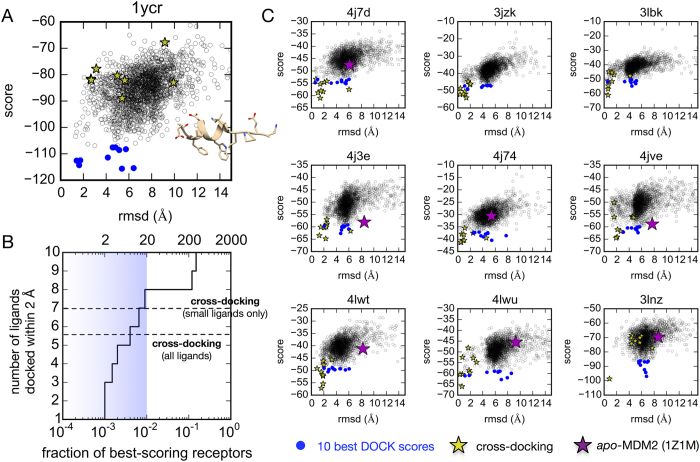
Scatter plots of DOCK scores versus the rmsd of the docked pose for all 2000 MSM receptor microstates show correlated funnel-like landscapes. (**A**) For the p53 ligand, the MSM receptor ensemble is more suitable for docking than any of the co-crystal receptor structures with other ligands. (**B**) The number of correct docking predictions found in some number of best-scoring poses (the true positive rate) for our test set is comparable to the cross-docking results. (**C**) Scatter plots for all ligands in our test set, shown with the 10 best-scoring poses docked to the MSM microstates (blue dots), cross-docking results (yellow stars), and docking results to the *apo*-MDM2 NMR structure (purple star, absences denote DOCK failures).

By comparison, computational docking to the ensemble of 2000 MSM microstates is much more successful. Plots of the DOCK score versus ligand pose rmsd show a funnel-like correlation, indicating that low scores indeed predict good ligand poses (Fig. 4). Because of this, a significant enrichment in correct docking predictions is achieved. If only the five best-scoring receptor poses were considered (the top 0.25%), half of the ligands would be correctly docked; 80% are correctly docked if only the top 20 (1%) receptor poses are considered.

A potential caveat of these results is that the DOCK energy function is designed for the inexpensive evaluation of very large screening sets, at the potential cost of accuracy. For the PMI N8A peptide ligand, the lowest-energy DOCK score consistently predicts a non-native pose in which the key tryptophan and phenylalanine are placed correctly in the binding site, but with non-native sidechain rotamers, turning the PMI helix ~30° in the binding cleft. We explored several alternative protocols designed to test whether this was due to our artificial cyclization scheme used to fix the backbone, or other search parameters; based on similar results in all cases, we conclude that scoring function accuracy is responsible.

### Simulation of functional lid motions is key to successful computational docking

**Figure 5:**
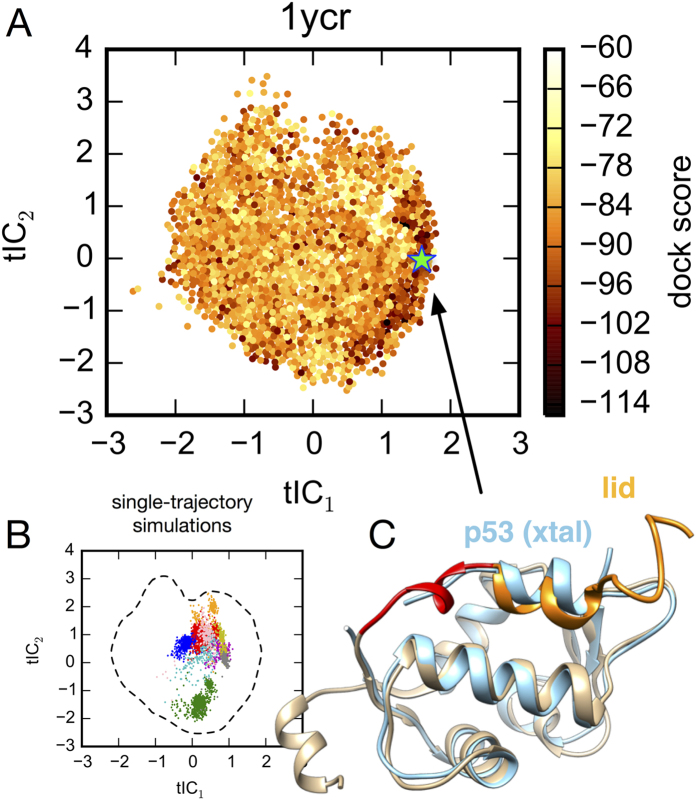
(**A**) Projection of the DOCK scores for p53 + MDM2 to the tICA landscape reveals a significant clustering of low-scoring poses corresponding to lid-associated structures. (**B**) Previous 200-ns and 1-*μ*s single-trajectory simulations of *apo*-MDM2 by Pantelopulos *et al*.^26^ projected to the tICA landscape show that simulations do not sample the full extent of lid motion seen in the MSM. Simulations were performed in force fields AMBER ff14sb (1 *μ*s, blue), ff99sb-ildn-nmr (1 *μ*s, red), ff99sb-ildn (200 ns, cyan), ff99sb (200 ns, yellow), ff99sb-ildn-phi (200 ns, orange), ff14sb (200 ns, magenta), CHARMM22* (1 *μ*s, green), CHARMM36 (200 ns, pink). (**C**) The receptor structure with the lowest DOCK score (green star, panel A) exhibits a lid conformation closely mimicking the structure of p53 TAD bound to MDM2.

Since our simulations started from an *apo*-MDM2 structure with a closed binding cleft not amenable to computational docking, we were curious to see how the functional lid motions identified in the MSM might be related to the generation of docking-competent receptor structures. A projection of the DOCK scores to the tICA landscape reveals that a significant clustering of low-scoring poses are found on the far right edge of the landscape, corresponding to states where the lid region is associated with the p53-binding cleft (Fig. 5a). This feature is more pronounced for the peptide docking results, but can also be seen clearly for the small-docking results (Figure S7). In previous work, we performed a number of *apo*-MDM2 simulations in various force fields, with trajectory lengths up to 1 *μ*s. The projection of these data onto the tICA landscapes shows that, regardless of the force field chosen, these simulations do not sample the full extent of lid motion seen in the MSM (Fig. 5b).

An inspection of the MDM2 receptor structures found on the far right of the tICA landscape, in the region of lowest DOCK scores, reveals many receptor conformations with their lid region associating with the MDM2 binding cleft. Indeed, the lowest-scoring receptor structure in this region for the p53 ligand (Fig. 5a, green star) is revealed to have a helical conformation, closely mimicking the bound pose of the p53 transactivation domain (Fig. 5c). In the unbound state, residues 11–17 (DGAVTTS) of the lid region have a low propensity to form a p53-like helix, forming helical structure when bound in the cleft (Figure S8).

## Discussion

### Comparison against previous experimental and computational results

The MDM2 lid region has been extensively studied experimentally and computationally. Here, we find that the results of our MSM are highly consistent with the accepted view of the structure and dynamics and of the lid region. NMR spectroscopy has determined the existence of two distinct conformational substates of the lid: a 90% population of *apo*-MDM2 is “closed”, with the lid region occluding the p53 binding cleft, while the remaining population is “open”^19^. In the *holo* state, the MDM2 lid is fully displaced by p53, in the “open” conformational state. These two states undergo slow (>10 ms) two-state exchange, with well defined peaks in chemical shifts indicating much faster conformational rearrangement within each conformational state. Our results agree well with this two-state picture, which is particularly remarkable because of the much shorter timescales of the simulation trajectories employed in our study. Despite these short (<1 *μs*) non-equilibrium timescales, MSM approaches are able to predict two-state conformational transitions, albeit on faster timescales. Recent accelerated MD studies of the lid region free energy landscape discern similar “open” and “closed” basins, as well as a “semi-open” basin^31^. As an important check on the accuracy of our simulation work, we show that our results quantitatively agree with NMR chemical shifts measured for the lid region. Although there are some mismatches in simulated versus experimental values, we attribute this to expected systematic inaccuracies of both the force field and the SHIFTX2 algorithm, as well as from the fact that truly slow processes (>10 ms) are not sampled in the simulations.

The MSM predictions are also remarkable for the extent of diffusivity and heterogeneity predicted for the “open” and “closed” lid states, which is in line expectations for intrinsically disordered peptides; indeed, previous MSMs constructed for disordered, aggregation-prone peptides show a distinct lack of structural intermediates^27^. The induced-fit “fly-casting” mechanism, in which intrinsically disordered peptides (including the p53 TAD of MDM2^17^) can fold upon binding, has been proposed as the dominant mechanism by which such peptides recognize their binding targets^32^. Lid region dynamics and cleft association could be classified similarly, although much of the lid remains unfolded. From the projections of computational docking scores to the tICA landscapes, it is clear that induction of binding-competent receptor structures is highly coupled to the two-state motion of lid association. We also note that previous 200-ns and 1-*μ*s simulations of *apo*-MDM2 starting from an initial closed-cleft NMR structure sample a range of open- and closed-cleft structures, but do not visit receptor structures highly competent for p53 binding, presumably because in these trajectories the lid region doesn’t sufficiently associate with the cleft to induce such structures.

### Implications for MSM methods in computational drug design

NMR spectroscopy shows that the binding of nutlin-3 to the MDM2 cleft preserves the “closed” state of the lid region^19^. Recent computational studies have examined how bound ligands (and/or post-translational modifications^33^) modulate the conformational dynamics of the lid region, with similar findings^31^. These authors also find that different lid conformations are preferred for different ligands, which, along with a growing number of published co-crystal structures with structured lid regions^2^,^20^, suggests that modeling the structure of the lid and its interaction with small-molecular inhibitors could lead to improved computational drug design.

A key question is whether such structural information could be obtained from *apo* state simulations, independent from the modeling of any particular bound ligand. Our computational docking results partially address this question by evaluating the quality of MSM-derived receptor structures, which were sampled in simulations where the lid region was included, but docked without the lid region. Several previous studies have notably performed computational docking to flexible receptor ensembles^34^,^35^,^36^,^37^, including MSM states derived from large-scale receptor simulations^38^,^39^,^40^. Kohlhoff *et al*. used the Surflex algorithm to dock ~8000 compounds from the ZINC library to MSM states of *β*_2_-adrenergic receptor, and found statistically significant enrichment predictions^39^. Our study is the first to compare the success rates of computational docking to MSMs to the “gold standard” of crystal structure cross-docking. We find that the top 1% of best-scoring MSM-derived receptor structures are highly “dockable” with an 80% true positive rate across our corpus of ligands, comparable to the success of cross-docking. These findings underscore the utility of large-scale conformational sampling and analysis made possible by Markov State Model approaches. In the future, MSMs are likely to be a valuable component of emerging molecular simulation-based methods for ensemble-based virtual screening^38^,^41^,^42^, especially for homology models^43^.

Given the known limitations in the accuracy of scoring functions for computational docking, we expect that the use of MD + MSM simulated receptor ensembles will perform even better in conjunction with more accurate energy functions, especially as a starting point for more sophisticated methods such as free energy perturbation^44^, for which elucidation of relative binding modes is especially important^45^.

Finally, we note that many drug targets are cell signaling proteins regulated in some way by intrinsically disordered binding partners. Many of these also have intrinsically disordered auto-inhibitory sequences than can mimic these natural substrates. For example, p53 binding partner MDMX was recently found to have an auto-inhibitory domain that inhibits binding through structural mimicry of the p53-MDMX interaction^46^, a discovery which helps explain the failure of prior small-molecule drug screening efforts that did not utilize the full-length target. Similarly, our results suggest that explicit consideration of such disordered regions in simulation models may be much more important than currently appreciated, and could lead to greater functional insights and more successful computational drug discovery efforts.

## Conclusion

Large-scale molecular simulation combined with Markov State Model analysis of simulated *apo*-MDM2 dynamics predicts diffuse, yet two-state binding of its disordered lid region to the p53 cleft, consistent with experiment. Computational docking of known MDM2 ligands to this simulated receptor ensemble achieves success comparable to crystal structure cross-docking, suggesting that virtual screening studies can benefit from Markov State Model approaches. These results underscore the importance of the disordered lid region in both understanding MDM2 functional motions and in computational drug discovery.

## Methods

### Molecular Simulation

GROMACS 4.5 was used for all simulation preparation and production^47^. Twenty-four initial conformations of the p53-binding region of *apo*-MDM2 (residues 1–119) were taken from the NMR-derived structural ensemble (PDB: 1Z1M)^48^. The AMBER ff99sb-ildn-nmr force field^49^ was chosen based on previous work demonstrating its accuracy and ability to predict initial structuring of the lid region in 1 *μ*s simulations^44^. All systems were constructed as periodic cubic boxes solvated with 17268 explicit TIP3P waters and 0.1 M NaCl. Stochastic (Langevin) dynamics was simulated using a leap-frog integrator with a time step of 2 fs and an inverse friction constant of 1 ps. Non-bonded cutoffs of 0.9 nm were used for both real-space Particle-Mesh Ewald (PME) electrostatic and vdW interactions. Protein and non-protein atoms were temperature- and pressure-coupled as separate groups in the Berendsen thermostat, at 300K and 1 atm, using a 1 ps time constant, compressibility of 4.5 × 10^−5^ bar^−1^. Prior to production runs, all systems were equilibrated in the isothermal-isobaric (NPT) ensemble until the system volume converged to 538.71 nm^3^. Production runs in the canonical (NVT) ensemble were performed on the Folding @ home distributed computing network^50^, obtaining 175.7 *μ*s of aggregate trajectory data. The distribution of trajectory lengths is roughly exponential, with a maximum trajectory length of 945 ns, and average trajectory length of 67.0 ns (Figure S1).

### Markov State Model (MSM) construction

MSMBuilder^51^ was used to construct MSMs from the trajectory data. Time-lagged independent component analysis (tICA) was performed using a tICA lag time of one snapshot (100 ps), to find a low-dimensional subspace best capturing the slowest motions of the N-terminus and its binding cleft. The subspace consists of linear combinations of the set of 2304 pairwise distances between all C_*α*_ atoms either in residues 1–24 of MDM2, or within 5 Å of any atom of the p53 helix in the crystal structure of *holo*-MDM2 (PDB: 1YCR). Conformational clustering in this low-dimensional subspace was used to define a set of 2000 metastable microstates. The generalized matrix Rayleigh quotient (GMRQ) method^52^ was used to find optimal MSM model parameters. This analysis, which involves a cross-validation procedure wherein the trajectory data is partitioned in testing and training sets, determined that (1) *k*-centers clustering produced marginally better models than *k*-means, (2) only two tICA components were needed to accurately capture the slowest conformational motions, (3) an MSM lag time of 100 ps produced the most accurate MSMs, and (4) the GMRQ score (reflecting model quality) plateaus around 2000 microstates (Figure S2). With metastable microstates suitably defined, the matrix of transition probabilities 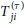 of transitioning from state *i* to state *j* within lag time *τ* was computed using a maximum-likelihood estimator from the observed transition counts^51^. Coarse-graining of MSM microstates into a 150-macrostate model was performed using the BACE algorithm^53^.

### Structural analysis

Analysis of trajectory data was performed using the MDTraj python library. Secondary structure populations were computed using the DSSP algorithm, with helical states corresponding to DSSP assignments G, H, I, and sheet states corresponded to DSSP assignments B and E. The SHIFTX2 algorithm^54^ was used to predict chemical shift values, using 10x subsampling of trajectory snapshots, for each MSM macrostate. To quantify the significance of interresidue contacts formed in specific conformational states, we compute a Bayes Factor (BF) contact metric for each residue pair in MDM2^25^. More details about this are given in the Supporting Information.

### Computational docking with DOCK6

Computational docking was performed using UCSF DOCK version 6.7^55^,^56^,^57^. The crystal structure coordinates were downloaded from the PDB and processed using the UCSF Chimera dockprep tool^57^,^58^. Small molecules were assigned AM1-BCC ligand partial charges with AmberTools antechamber^59^, while peptide ligands were assigned ff14SB charges. Frames taken from each of the 2000 microstate clusters were converted into DOCK-compatible MOL2 files. Owing to inconsistencies in hydrogen atom naming schemes, each such frame was reassigned optimized instantaneous protonation states using the REDUCE tool^60^. Grids at 0.3 Å-resolution were computed for each of the 2000 MD-derived frames. In order to improve sampling, each rigid segment with five or more atoms (e.g. pyrroles or larger) was used as an anchor during small molecule docking. A unique feature of the DOCK program is the anchor and grow algorithm^55^. A rigid section of the molecule, often a large aromatic scaffold (anchor) is first oriented in the binding site. The remaining torsions are then grown one-by-one, clustering and pruning unfavorable conformations at every step until a final set of viable fully grown conformers remain. This breadth-first search approach takes exponential computational time, which severely limits docking of larger molecules. DOCK 5 was only tested on a set of molecules with seven or fewer rotatable bonds^61^. For DOCK 6.2 onwards, the addition of a fast internal energy score, coupled with aggressive pruning and rmsd symmetry, allowed reasonable performance with larger molecules (65.5% success on 8–15 torsions and 48% with >15 torsions)^55^. Earlier work^55^ demonstrated that despite these gains, docking success drops linearly with the number of rotatable bonds, while runtime increases exponentially^62^,^63^.

DOCK considers closed cycles in molecules to be rigid when sampling torsions. However, the simplex minimizer still relaxes local backbone conformations within these cycles. Thus, for peptide ligands, we introduced an artificial bond between the N- and C-termini to rigidify the backbone for the purposes of docking. This ameliorates the need to fold alpha helical ligands *ab initio* with the limited molecular mechanics scoring function van der Waals and electrostatics with a distance dependent dielectric) in DOCK. In the case of the p53 TAD fragment, this reduces 66 torsions to 29 torsions after rigidifying the backbone. DOCK thus considers the backbone to be an anchor, with each sidechain torsion grown *in situ* for each receptor microstate. Cases where the receptor conformation does not (1) accommodate the backbone, or (2) allow all the sidechains to complete growth, forces the docked ligand out of the binding site, resulting in a poor interaction score.

## Additional Information

**How to cite this article**: Mukherjee, S. *et al*. Markov models of the *apo*-MDM2 lid region reveal diffuse yet two-state binding dynamics and receptor poses for computational docking. *Sci. Rep.* **6**, 31631; doi: 10.1038/srep31631 (2016).

## Supplementary Material

Supplementary Information

## Figures and Tables

**Figure 1:**
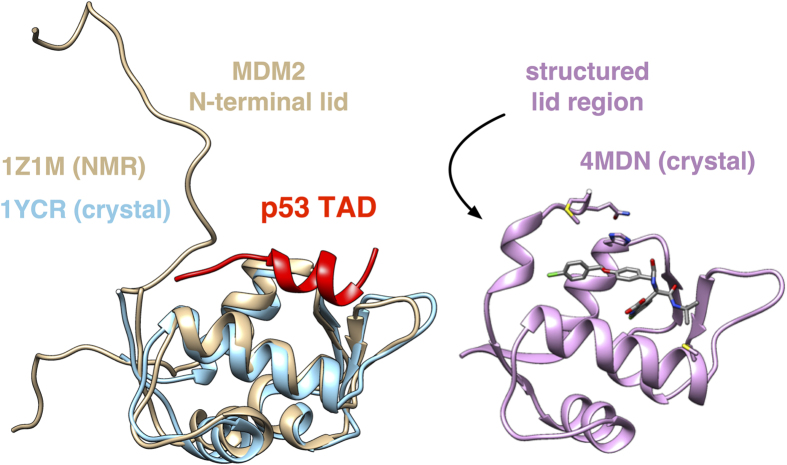
*Apo* and *holo* structures of MDM2. (left) The *apo* form of MDM2 (tan) has an unstructured N-terminal lid (residues 1–25) that associates with the cleft. The binding of the p53 transactivation domain (TAD, red) displaces the lid region from the binding cleft, as seen in the *holo* crystal structure (blue). Quantitative NMR of *apo*-MDM2^19^ has shown that a portion of the lid region (residues 19–24) slowly converts between an unstructured and a structured state. A recent co-crystal structure^20^ with a small-molecule inhibitor (pink) shows a structured form of the lid region.

**Table 1:** Test set of small-molecule and peptide ligands of MDM2 used for computational docking studies.

Molecule	PDB	Ligand	Rot. bonds	Affinity (nM)	Reference
1	4j7d	Nutlin RO5045331	6	20000	Fry, *et al*. ACS Med Chem Lett 2013
2	3jzk	chromeno-triazolo-pyrimidine	2	1230	Allen, *et al*. J Med Chem 2009
3	3lbk	WK23	3	916	Popowic, *et al*. Cell Cycle 2010
4	4j3e	Nutlin 3a	8	88	Vu, *et al*. ACS Med Chem Lett 2013
5	4j74	nutlin RO0503918	2	26000	Fry, *et al*. ACS Med Chem Lett 2013
6	4jve	morpholinone 16	8	86	de Turiso, *et al*. J Med Chem 2013
7	4lwt	spiroindolinone RO5027344	4	3900	Zhang, *et al*. Bioorg Med Chem 2014
8	4lwu	spiroindolinone RO5499252	8	5	Zhang, *et al*. Bioorg Med Chem 2014
9*	1ycr	p53 (ETFSDLWKLLPE)	29	440	Kussie, *et al*. Science 1996
10*	3lnz	N8A PMI (TSFAEYWALLSP)	21	0.49	Li *et al*. JMB 2010

^*^Peptides.

## References

[b1] Zhao Y., Aguilar A., Bernard D. & Wang S. Small-molecule inhibitors of the mdm2–p53 protein–protein interaction (mdm2 inhibitors) in clinical trials for cancer treatment. J. Med. Chem. 58, 1038–1052 (2015). 25396320 10.1021/jm501092z PMC4329994

[b2] Estrada-Ortiz N., Neochoritis C. G. & Dömling A. How to design a successful p53-mdm2/x interaction inhibitor: A thorough overview based on crystal structures. Chem. Med. Chem. (2015). 10.1002/cmdc.201500487 PMC4838565 26676832

[b3] Rew Y. . Structure-based design of novel inhibitors of the mdm2-p53 interaction. J. Med. Chem. 55, 4936–4954 (2012). 22524527 10.1021/jm300354j

[b4] Baek S. . Structure of the stapled p53 peptide bound to mdm2. J. Am. Chem. Soc. 134, 103–106 (2012). 22148351 10.1021/ja2090367

[b5] Kritzer J. A., Lear J. D., Hodsdon M. E. & Schepartz A. Helical *β*-peptide inhibitors of the p53-hdm2 interaction. J. Am. Chem. Soc. 126, 9468–9469 (2004). 15291512 10.1021/ja031625a

[b6] Brown Z. Z. . A spiroligomer *α*-helix mimic that binds hdm2, penetrates human cells and stabilizes hdm2 in cell culture. PLoS One 7, e45948 (2012). 23094022 10.1371/journal.pone.0045948 PMC3475717

[b7] Liu M. . A left-handed solution to peptide inhibition of the p53-mdm2 interaction. Angew. Chem. Int. Ed. 49, 3649–3652 (2010). 10.1002/anie.201000329 PMC3359147 20449836

[b8] Pazgier M. . Structural basis for high-affinity peptide inhibition of p53 interactions with mdm2 and mdmx. Proc. Natl. Acad. Sci. USA 106, 4665–4670 (2009). 19255450 10.1073/pnas.0900947106 PMC2660734

[b9] Zhan C. . An ultrahigh affinity d-peptide antagonist of mdm2. J. Med. Chem. 55, 6237–6241 (2012). 22694121 10.1021/jm3005465 PMC3426300

[b10] Henchey L. K., Jochim A. L. & Arora P. S. Contemporary strategies for the stabilization of peptides in the *α*-helical conformation. Curr. Opin. Chem. Biol. 12, 692–697 (2008). 18793750 10.1016/j.cbpa.2008.08.019 PMC2650020

[b11] Lao B. B. . Rational design of topographical helix mimics as potent inhibitors of protein-protein interactions. J. Am. Chem. Soc. 136, 7877–7888 (2014). 24972345 10.1021/ja502310r PMC4353027

[b12] Michel J., Harker E. A., Tirado-Rives J., Jorgensen W. L. & Schepartz A. In silico improvement of *β* 3-peptide inhibitors of p53•hdm2 and p53•hdmx. J. Am. Chem. Soc. 131, 6356–6357 (2009). 19415930 10.1021/ja901478e PMC2754742

[b13] Fuller J. C., Jackson R. M., Edwards T. A., Wilson A. J. & Shirts M. R. Modeling of arylamide helix mimetics in the p53 peptide binding site of hdm2 suggests parallel and anti-parallel conformations are both stable. PLoS One 7, e43253 (2012). 22916232 10.1371/journal.pone.0043253 PMC3423354

[b14] Fuller J. C., Jackson R. M. & Shirts M. R. Configurational preferences of arylamide *α*-helix mimetics via alchemical free energy calculations of relative binding affinities. J. Phys. Chem. B 116, 10856–10869 (2012). 22920218 10.1021/jp209041x

[b15] ElSawy K. M., Lane D. P., Verma C. S. & Caves L. S. D. Recognition dynamics of p53 and mdm2: Implications for peptide design. J. Phys. Chem. B 120, 320–328 (2016). 26701330 10.1021/acs.jpcb.5b11162

[b16] Guo Z., Streu K., Krilov G. & Mohanty U. Probing the origin of structural stability of single and double stapled p53 peptide analogs bound to mdm2. Chem. Biol. Drug Des. 83, 631–642 (2014). 24418072 10.1111/cbdd.12284

[b17] Wright P. E. & Dyson H. J. Intrinsically disordered proteins in cellular signalling and regulation. Nat. Rev. Mol. Cell Biol. 16, 18–29 (2015). 25531225 10.1038/nrm3920 PMC4405151

[b18] Borcherds W. . Disorder and residual helicity alter p53-mdm2 binding affinity and signaling in cells. Nat. Chem. Biol. 1–5 (2014). 10.1038/nchembio.1668 25362358

[b19] Showalter S. A., Bruschweiler-Li L., Johnson E., Zhang F. & Brüschweiler R. Quantitative lid dynamics of mdm2 reveals differential ligand binding modes of the p53-binding cleft. J. Am. Chem. Soc. 130, 6472–6478 (2008). 18435534 10.1021/ja800201j

[b20] Bista M. . Transient protein states in designing inhibitors of the mdm2-p53 interaction. Structure 21, 2143–2151 (2013). 24207125 10.1016/j.str.2013.09.006 PMC4104591

[b21] Chodera J. D. & Noé F. Markov state models of biomolecular conformational dynamics. Curr. Opin. Struct. Biol. 25, 135–144 (2014). 24836551 10.1016/j.sbi.2014.04.002 PMC4124001

[b22] Schwantes C. R. & Pande V. S. Improvements in markov state model construction reveal many non-native interactions in the folding of ntl9. J. Chem. Theor. Comput. 9, 2000–2009 (2013). 10.1021/ct300878a PMC3673732 23750122

[b23] Perez-Hernandez G., Paul F., Giorgino T., De Fabritiis G. & Noé F. Identification of slow molecular order parameters for markov model construction. J. Chem. Phys. 139, 015102 (2013). 23822324 10.1063/1.4811489

[b24] Razavi A. M. & Voelz V. A. Kinetic network models of tryptophan mutations in *β*-hairpins reveal the importance of non-native interactions. J. Chem. Theor. Comput. 11, 2801–2812 (2015). 10.1021/acs.jctc.5b00088 26575573

[b25] Zhou G. & Voelz V. A. Using kinetic network models to probe non-native salt-bridge effects on *α*-helix folding. J. Phys. Chem. B 120, 926–935 (2016). 26769494 10.1021/acs.jpcb.5b11767 PMC7600393

[b26] Boninsegna L., Gobbo G., Noé F. & Clementi C. Investigating molecular kinetics by variationally optimized diffusion maps. J. Chem. Theor. Comput. 11, 5947–5960 (2015). 10.1021/acs.jctc.5b00749 26580713

[b27] Lin Y.-S., Bowman G. R., Beauchamp K. A. & Pande V. S. Investigating how peptide length and a pathogenic mutation modify the structural ensemble of amyloid beta monomer. Biophys. J. 102, 315–324 (2012). 22339868 10.1016/j.bpj.2011.12.002 PMC3260686

[b28] Qiao Q., Bowman G. R. & Huang X. Dynamics of an intrinsically disordered protein reveal metastable conformations that potentially seed aggregation. J. Am. Chem. Soc. 135, 16092–16101 (2013). 24021023 10.1021/ja403147m

[b29] Kussie P. H. . Structure of the mdm2 oncoprotein bound to the p53 tumor suppressor transactivation domain. Science 274, 948–953 (1996). 8875929 10.1126/science.274.5289.948

[b30] Li C. . Systematic mutational analysis of peptide inhibition of the p53–mdm2/mdmx interactions. J. Mol. Biol. 398, 200–213 (2010). 20226197 10.1016/j.jmb.2010.03.005 PMC2856455

[b31] Bueren-Calabuig J. A. & Michel J. Elucidation of ligand-dependent modulation of disorder-order transitions in the oncoprotein mdm2. PLoS Comput. Biol. 11, e1004282 (2015). 26046940 10.1371/journal.pcbi.1004282 PMC4457491

[b32] Sugase K., Dyson H. J. & Wright P. E. Mechanism of coupled folding and binding of an intrinsically disordered protein. Nature 447, 1021–1025 (2007). 17522630 10.1038/nature05858

[b33] Bueren-Calabuig J. A. & Michel J. Impact of ser17 phosphorylation on the conformational dynamics of the oncoprotein mdm2. Biochemistry 55, 2500–2509 (2016). 27050388 10.1021/acs.biochem.6b00127

[b34] Amaro R. E. & Li W. W. Emerging Methods for Ensemble-Based Virtual Screening. Curr. Top. Med. Chem. 10, 3–13 (2010). 19929833 10.2174/156802610790232279 PMC3086266

[b35] Feixas F., Lindert S., Sinko W. & McCammon J. A. Exploring the role of receptor flexibility in structure-based drug discovery. Biophys. Chem. 186, 31–45 (2014). 24332165 10.1016/j.bpc.2013.10.007 PMC4459653

[b36] Fischer M., Coleman R. G., Fraser J. S. & Shoichet B. K. Incorporation of protein flexibility and conformational energy penalties in docking screens to improve ligand discovery. Nat. Chem. 6, 575–583 (2014). 24950326 10.1038/nchem.1954 PMC4144196

[b37] Wagner J. R., Lee C. T., Durrant J. D., Malmstrom R. D., Feher V. A. & Amaro R. E. Emerging Computational Methods for the Rational Discovery of Allosteric Drugs. Chem. Rev. 116, 6370–6390 (2016). 27074285 10.1021/acs.chemrev.5b00631 PMC4901368

[b38] Shukla D., Hernández C. X., Weber J. K. & Pande V. S. Markov state models provide insights into dynamic modulation of protein function. Acc. Chem. Res. 48, 414–422 (2015). 25625937 10.1021/ar5002999 PMC4333613

[b39] Kohlhoff K. J., Shukla D., Lawrenz M., Bowman G. R., Konerding D. E., Belov D., Altman R. B. & Pande V. S. Cloud-based simulations on Google Exacycle reveal ligand modulation of GPCR activation pathways. Nat. Chem. 6, 15–21 (2014). 24345941 10.1038/nchem.1821 PMC3923464

[b40] Bowman G. R., Bolin E. R., Kathryn M. H., Maguire B. C. & Marqusee S. Discovery of multiple hidden allosteric sites by combining Markov state models and experiments. Proc. Natl. Acad. Sci. USA 112, 2734–2739 (2015). 25730859 10.1073/pnas.1417811112 PMC4352775

[b41] Cheng L. S. . Ensemble-based virtual screening reveals potential novel antiviral compounds for avian influenza neuraminidase. J. Med. Chem. 51, 3878–3894 (2008). 18558668 10.1021/jm8001197 PMC2652358

[b42] Malmstrom R. D., Lee C. T., Van Wart A. T. & Amaro R. E. Application of molecular-dynamics based markov state models to functional proteins. J. Chem. Theor. Comput. 10, 2648–2657 (2014). 10.1021/ct5002363 PMC4248791 25473382

[b43] Choi J., Choi K.-E., Park S. J., Kim S. Y. & Jee J.-G. Ensemble-based virtual screening led to the discovery of new classes of potent tyrosinase inhibitors. J. Chem. Inf. Model. 56, 354–367 (2016). 26750991 10.1021/acs.jcim.5b00484

[b44] Pantelopulos G. A., Mukherjee S. & Voelz V. A. Microsecond simulations of mdm2 and its complex with p53 yield insight into force field accuracy and conformational dynamics. Proteins: Struct., Funct., Bioinf. 83, 1665–1676 (2015). 10.1002/prot.24852 26138282

[b45] Mobley D. L. & Klimovich P. V. Perspective: Alchemical free energy calculations for drug discovery. J. Chem. Phys. 137, 230901 (2012). 23267463 10.1063/1.4769292 PMC3537745

[b46] Chen L. . Autoinhibition of mdmx by intramolecular p53 mimicry. Proc. Natl. Acad. Sci. USA 112, 4624–4629 (2015). 25825738 10.1073/pnas.1420833112 PMC4403185

[b47] Pronk S. . Gromacs 4.5: a high-throughput and highly parallel open source molecular simulation toolkit. Bioinformatics 29, 845–854 (2013). 23407358 10.1093/bioinformatics/btt055 PMC3605599

[b48] Uhrinova S. . Structure of free mdm2 n-terminal domain reveals conformational adjustments that accompany p53-binding. J. Mol. Biol. 350, 587–598 (2005). 15953616 10.1016/j.jmb.2005.05.010

[b49] Li D.-W. & Brüschweiler R. Nmr-based protein potentials. Angew. Chem., Int. Ed. 49, 6778–6780 (2010). 10.1002/anie.201001898 20715028

[b50] Shirts M. & Pande V. S. Screen savers of the world, unite! Science 290, 1903–1904 (2000). 17742054 10.1126/science.290.5498.1903

[b51] Beauchamp K. A. . Msmbuilder2: Modeling conformational dynamics on the picosecond to millisecond scale. J. Chem. Theor. Comput. 7, 3412–3419 (2011). 10.1021/ct200463m PMC3224091 22125474

[b52] McGibbon R. T. & Pande V. S. Variational cross-validation of slow dynamical modes in molecular kinetics. J. Chem. Phys. 142, 124105 (2015). 25833563 10.1063/1.4916292 PMC4398134

[b53] Bowman G. R. Improved coarse-graining of markov state models via explicit consideration of statistical uncertainty. J. Chem. Phys. 137, 134111 (2012). 23039589 10.1063/1.4755751 PMC3477182

[b54] Han B., Liu Y., Ginzinger S. W. & Wishart D. S. Shiftx2: significantly improved protein chemical shift prediction. J. Biomol. NMR 50, 43–57 (2011). 21448735 10.1007/s10858-011-9478-4 PMC3085061

[b55] Allen W. J. . Dock 6: Impact of new features and current docking performance. J. Comp. Chem. 36, 1132–1156 (2015). 25914306 10.1002/jcc.23905 PMC4469538

[b56] Brozell S. R. . Evaluation of dock 6 as a pose generation and database enrichment tool. J. Comput.-Aided Mol. Des. 26, 749–773 (2012). 22569593 10.1007/s10822-012-9565-y PMC3902891

[b57] Lang P. T. . Dock 6: combining techniques to model rna-small molecule complexes. RNA 15, 1219–1230 (2009). 19369428 10.1261/rna.1563609 PMC2685511

[b58] Pettersen E. F. . UCSF Chimera-a visualization system for exploratory research and analysis. J. Comp. Chem. 25, 1605–1612 (2004). 15264254 10.1002/jcc.20084

[b59] Case C. . Amber 14. University of California, San Francisco (2015).

[b60] Word J. M., Lovell S. C., Richardson J. S. & Richardson D. C. Asparagine and glutamine: using hydrogen atom contacts in the choice of side-chain amide orientation. J. Mol. Biol. 285, 1735–1747 (1999). 9917408 10.1006/jmbi.1998.2401

[b61] Moustakas D. T. . Development and validation of a modular, extensible docking program: Dock 5. J. Comput.-Aided Mol. Des. 20, 601–619 (2006). 17149653 10.1007/s10822-006-9060-4

[b62] Nüske F., Keller B. G., Perez-Hernandez G., Mey A. S. J. S. & Noé F. Variational Approach to Molecular Kinetics. J. Chem. Theor. Comput. 10, 1739–1752 (2014). 10.1021/ct4009156 26580382

[b63] Noé F. & Clementi C. Kinetic Distance and Kinetic Maps from Molecular Dynamics Simulation. J. Chem. Theor. Comput. 11, 5002–5011 (2015). 10.1021/acs.jctc.5b00553 26574285

