# Supplementary material for: Markov models of the apo-MDM2 lid region reveal diffuse yet two-state binding dynamics and receptor poses for computational docking
Source: Sci Rep. 2016 Aug 19;6:31631. doi: 10.1038/srep31631 (PMC4990920; doi:10.1038/srep31631)
Supplement: Supplementary Information [file srep31631-s1.pdf]

Supplementary Information

Markov models of the *apo*-MDM2 lid region reveal diffuse yet two-state binding dynamics and receptor poses for computational docking

Sudipto Mukherjee, George A. Pantelopulos and Vincent A. Voelz
Department of Chemistry, Temple University, Philadelphia, PA, USA

Supporting Text

Generalized Matrix Raleigh Quotient (GMRQ) Analysis

To choose optimal parameters for MSM construction, we use the GMRQ method recently developed by McGibbon et al.[1] This method exploits the variational principle of conformational dynamics,[2–4], that any approximation to the true eigenvectors of a dynamical operator will necessarily underestimate its eigenvalues, which correspond to the relaxation timescales. For an MSM of n discrete conformational states indexed by $k = 1...n$, approximations to the eigenfunctions are linear combinations of indicator basis set functions $\phi_k(x)$ (equal to 1 if x is in state k and 0 otherwise.) The generalized matrix Raleigh quotient R quantifies how well a given linear combination of basis functions captures the m slowest eigenvectors/timescales. It is computed as

$$R(A|C,S) \equiv Tr(\frac{A^T CA}{A^T SA}) \tag{1}$$

where A is an $n \times m$ matrix whose j^{th} column contains the linear coefficients to approximate the jth eigenvector as $\sum_k A_{kj}\phi_k(x)$, C is an $n \times n$ time-lagged correlation matrix of the indicator basis functions calculated from the trajectory data, and S is an $n \times n$ covariance matrix of the indicator basis functions. For an MSM, both C and S can be estimated from the number of transitions between states observed in the simulation trajectories. Finding the coefficient matrix A that maximizes R is the same eigenproblem solved in the tICA method.[1] Once maximized, R can be used to evaluate the quality of the MSM state decomposition by how well it captures the true conformational dynamics.

To avoid overfitting to the data, a cross-validation approach is used in which the simulation data is partitioned into 5-fold leave-one-out training/testing sets. In five separate trials, R is maximized using 4/5 of the trajectory data, which we report as the GMRQ training score. Using these optimal values of the coefficients in A, the remaining 1/5 of the data is then used to compute R, which we report as the GMRQ testing score.

To select the MSM that most accurately captures the conformational dynamics of the MDM2 lid region, we explored various model construction parameters and chose the model with the largest GMRQ testing score (Figure S2). The time step between collected trajectory snapshots is τ_0 = 100 ps. We explored MSMs built using different tICA lag times (i.e. the lag time to calculate the distance correlation matrix needed to find the tICA components) and different MSM lag times. For MSMs constructed using a range of 200 to 2000 microstates, a lag time of 1 (in units of τ_0) is optimal (Figure S2a,b). We also explored MSMs constructed using different numbers of tICA components (2 to 15 tICs) as the subspace to project trajectory data and perform conformational clustering. We found that, for MSMs constructed using a range of 200 to 2000 microstates, projections utilizing 2 tICs give the best GMRQ testing scores (Figure S2c). In all of our tests, GMRQ testing scores versus the number of MSM microstates plateau near 2000 microstates, so we chose this number of microstates for MSM construction.

Given that typical MSM lag times for protein folding and binding studies range from 1-200 ns, it is somewhat surprising that such a short lagtime (100 ps) is preferred for MSM construction. To validate this result, we additionally built MSMs using lag times of 2, 5, 10, 100 and 1000 (in units of τ_0). We find that the equilibrium populations and slowest relaxation mode eigenvector ϕ_1 are remarkably robust at all lag times, with the exception of 1000, for which finite sampling artifacts become pronounced (Figure S3a). A likely explanation for the short lag time being optimal is the highly diffusive nature of the lid region dynamics, coupled with a trajectory data set enriched in many short simulations (see Figure S1). To test this idea, we built MSMs using lag times of 1, 10, 100 and 1000 using the same construction parameters, but without using a standard ergodic trimming step, which is usually employed to avoid statistical bias from non-equilibrium trajectories.[5] This bias is particularly pronounced for MSMs built from distributed computing simulations, due to the use of many short trajectories that make forward transitions to new states, but no backward transitions. Enforcing detailed balance on a MSM built from this data can therefore introduce "trap" artifacts in which states can have incorrectly high population estimates. Indeed, without ergodic trimming, the tICA model strongly exhibits the presence of traps, indicating the non-ergodicity of the underlying trajectory data arising from diffusivity and trajectory length (Figure S3b).

Bayes Factor analysis of inter-residue contacts

To quantify the significance of inter-residue contacts formed in specific conformational states, we compute a Bayes Factor (BF) contact metric for each residue pair in MDM2.[6] The $BF_k(i,j)$ for contacts between residues i and j, given the protein is in

some conformational state k, is computed as

$$BF_k(i,j) = \frac{P(k|c_{ij}=1)}{P(k|c_{ij}=0)} = \frac{(P(c_{ij}=1|k)}{P(c_{ij}=1)} \frac{P(c_{ij}=0)}{P(c_{ij}=0|k)}. \quad (2)$$

Here, c_{ij} is an indicator variable that takes the value of 1 if a contact between residues i and j is present, and 0 otherwise. The $BF_k(i,j)$ value can be thought as the statistical over-representation of contact c_{ij} in conformational state k, and hence a measure of its importance in uniquely defining the structural features of that state. For example, if $BF_k = 2$, that means that the equilibrium constant for contact formation between residues i and j is twice as large for state k as it is for the whole ensemble. We compute Bayes factors for contacts separated by three or more residues in sequence, and define a contact formed between two residues if any two non-hydrogen atoms are closer than 4 Å.

Supporting Figures

- Figure S1. Trajectory length distributions
- Figure S2. GMRQ Analysis
- Figure S3. Effects of lag time and/or ergodic trimming on constructed MSMs
- Figure S4. Free energy landscape of simulation data projected to tIC_1 and tIC_2
- Figure S5. Changes in inter-residue contacts and secondary structure along eigenmode relaxations ϕ_1 and ϕ_2.
- Figure S6. Bayes Factors of inter-residue contacts for tICA landscape quadrants.
- Figure S7. DOCK scores projected onto the 2D tICA landscape for all ligands
- Figure S8. Backbone RMSDs of MDM2 lid residues 11-17 to bound-state p53 helix.

References

1. McGibbon, R. T. & Pande, V. S. Variational cross-validation of slow dynamical modes in molecular kinetics. *The Journal of Chemical Physics* **142**, 124105 (2015). URL http://scitation.aip.org/content/aip/journal/jcp/142/12/10.1063/1.4916292.

2. Nüske, F., Keller, B. G., Perez-Hernandez, G., Mey, A. S. J. S. & Noé, F. Variational Approach to Molecular Kinetics. *Journal of Chemical Theory and Computation* **10**, 1739–1752 (2014).

3. Noé, F. & Clementi, C. Kinetic Distance and Kinetic Maps from Molecular Dynamics Simulation. *Journal of Chemical Theory and Computation* **11**, 5002–5011 (2015).

4. Boninsegna, L., Gobbo, G., Noé, F. & Clementi, C. Investigating Molecular Kinetics by Variationally Optimized Diffusion Maps. *Journal of Chemical Theory and Computation* **11**, 5947–5960 (2015).

5. Scalco, R. & Caflisch, A. Equilibrium Distribution from Distributed Computing (Simulations of Protein Folding). *The Journal of Physical Chemistry B* **115**, 6358–6365 (2011).

6. Zhou, G. & Voelz, V. A. Using kinetic network models to probe non-native salt-bridge effects on α-helix folding. *Journal of Physical Chemistry B* **120**, 926–935 (2016).

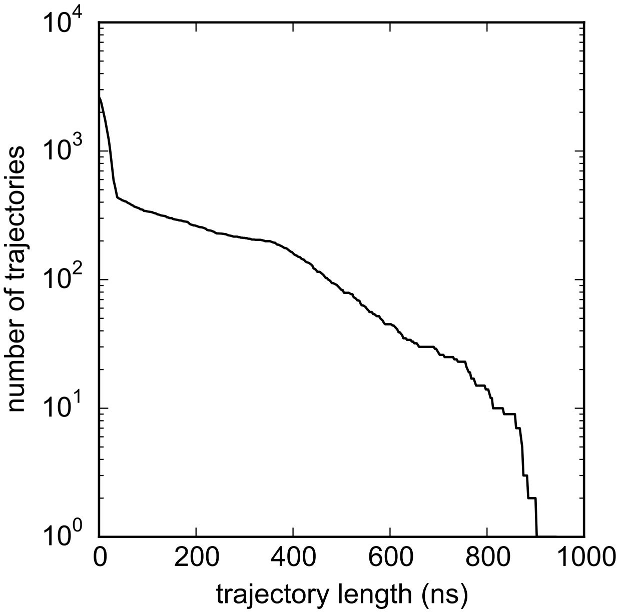

Figure S1. Distribution of trajectory lengths for the 175.7 μs of aggregate trajectory data simulated on the Folding@home distributed computing network. The maximum trajectory length is 945 ns, and average trajectory length is 67.0 ns.

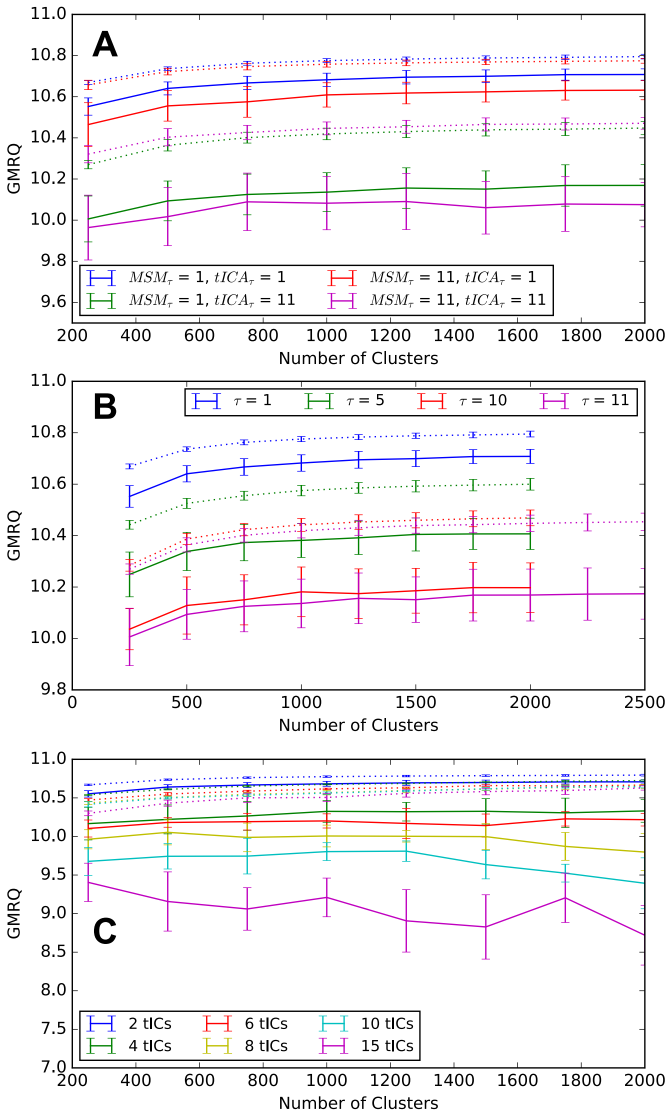

Figure S2. Mean GMRQ training scores (dotted line) and testing scores (solid line) computed using 5-fold cross validation, shown as a function of the number of *k*-centers clusters used to build the MSM. (A) GMRQ scores calculated using slow ($11\tau_0$) and fast ($1\tau_0$) MSM and tICA lag times. (B) GMRQ scores calculated using a range ($2\tau_0$ to $11\tau_0$) of MSM lag times; each MSM was constructed using clustering in the subspace defined by the 2 largest tICA components. (C) GMRQ scores calculated for MSMs built from various numbers of tICA components (2 to 15). Error bars in all figures show standard deviations of the 5-fold cross-validation trials.

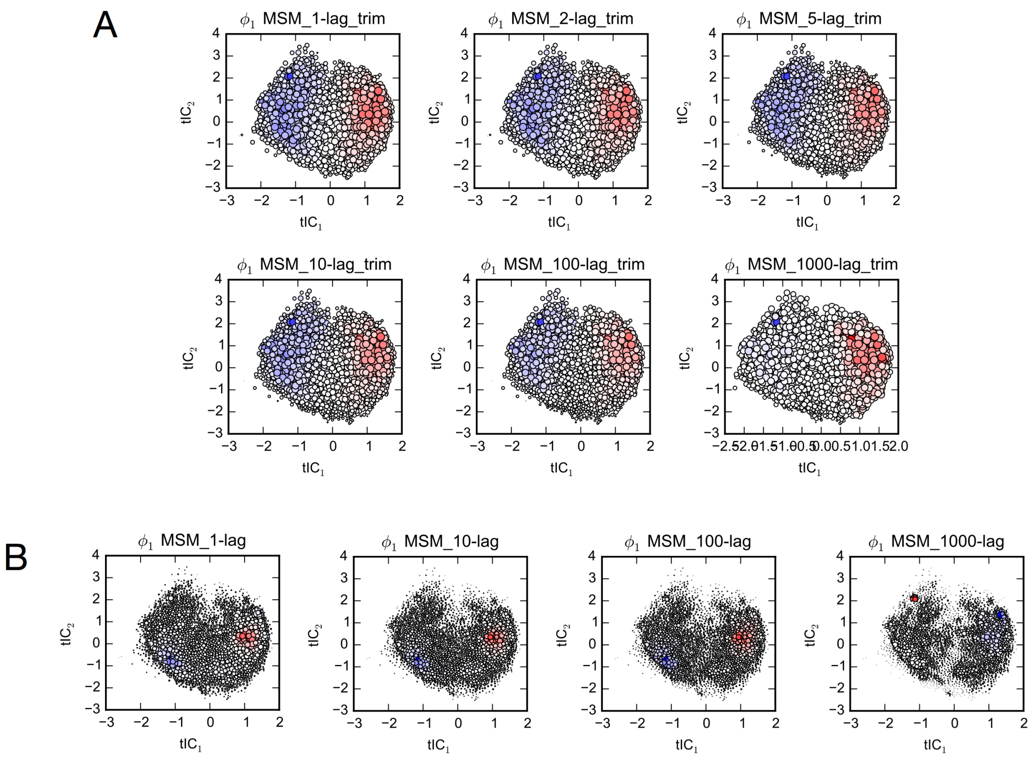

Figure S3. (A) Projections of the 2000 MSM microstates (filled circles) to tIC_1 and tIC_2 coordinates from MSMs built using lag times 1, 2, 5, 10, 100 and 1000 (in units τ_0). The size of each circle is proportional to the logarithm of its equilibrium population, and is colored according to the slowest relaxation eigenmode, ϕ_1. (B) MSMs built using lag times 1, 10, 100, 1000, without the standard ergodic trimming step.

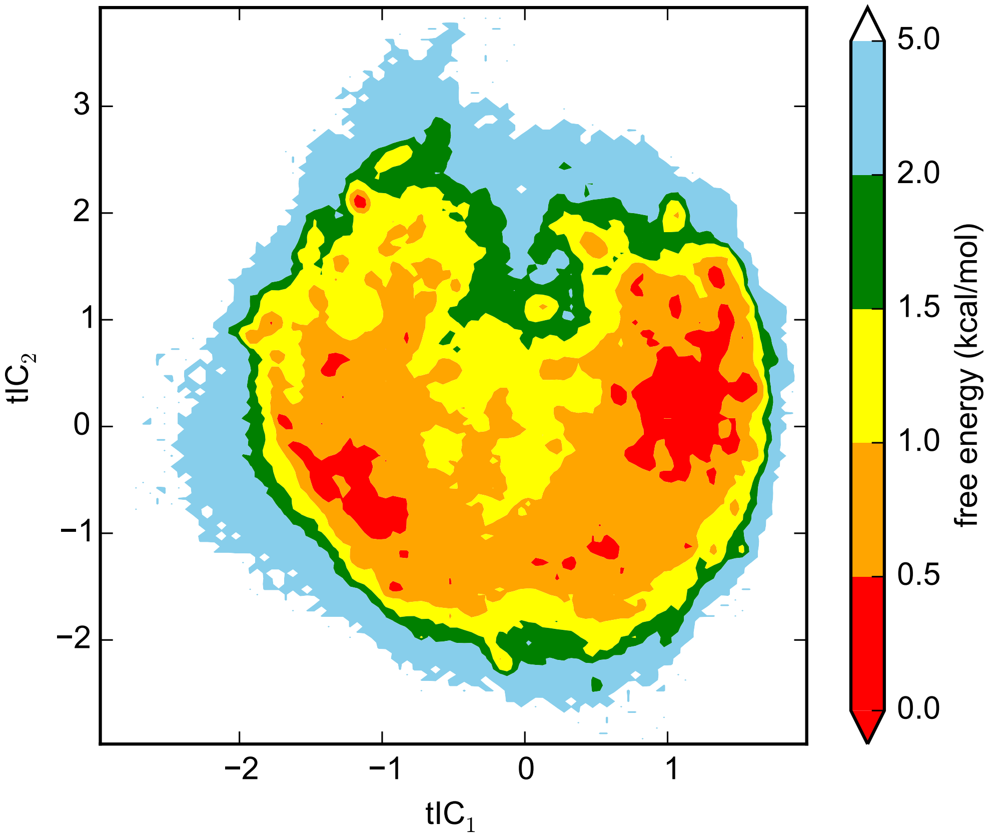

Figure S4. Projection of the simulation trajectory data to the tIC$_1$ and tIC$_2$ landscape.

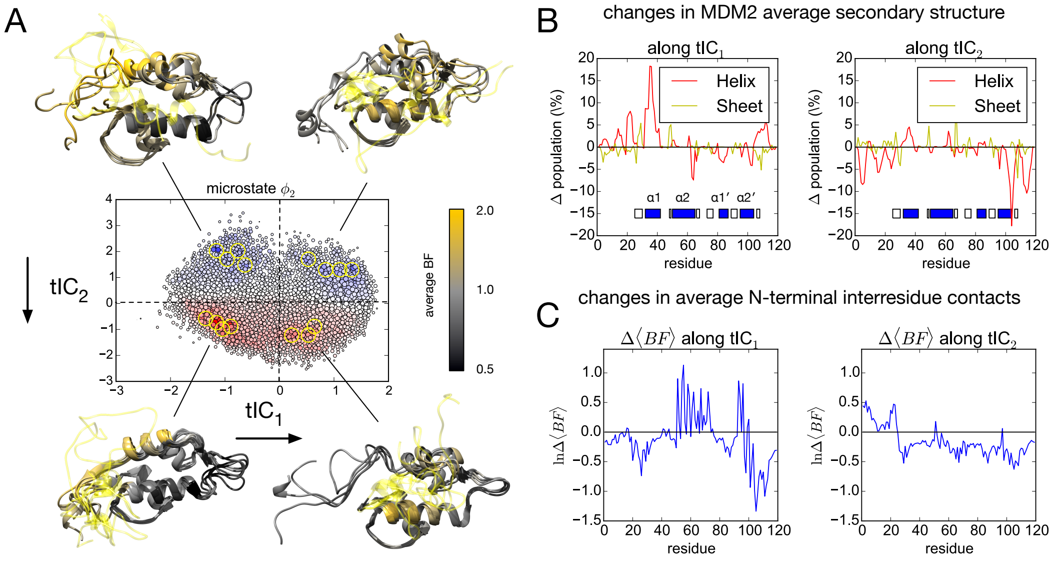

Figure S5. (A) Projection of the 2000 MSM microstates (filled circles) to tIC_1 and tIC_2 coordinates. The size of each circle is proportional to the logarithm of the equilibrium population, and is colored according to the second slowest relaxation eigenmode, ϕ_2. Population flux along this mode is from blue to red, predominantly representing the structuring of the MDM2 termini, which we visualize using five representative structures from each basin (circled in yellow on the tICA landscape). Average Bayes Factors for contacts between lid region (yellow ribbon) and non-lid residues are shown as a color gradient (black to orange) on the ribbon structure of MDM2. (B) Differences in per-residue helix and sheet content of MDM2 are shown along tIC_1 (i.e. differences between conformations with positive vs. negative values of tIC_1) and along tIC_2. (C) Changes in the ratio of mean Bayes Factors for contacts between the lid region (residues 1–25 of MDM2) and all other residues, along tIC_1 and tIC_2.

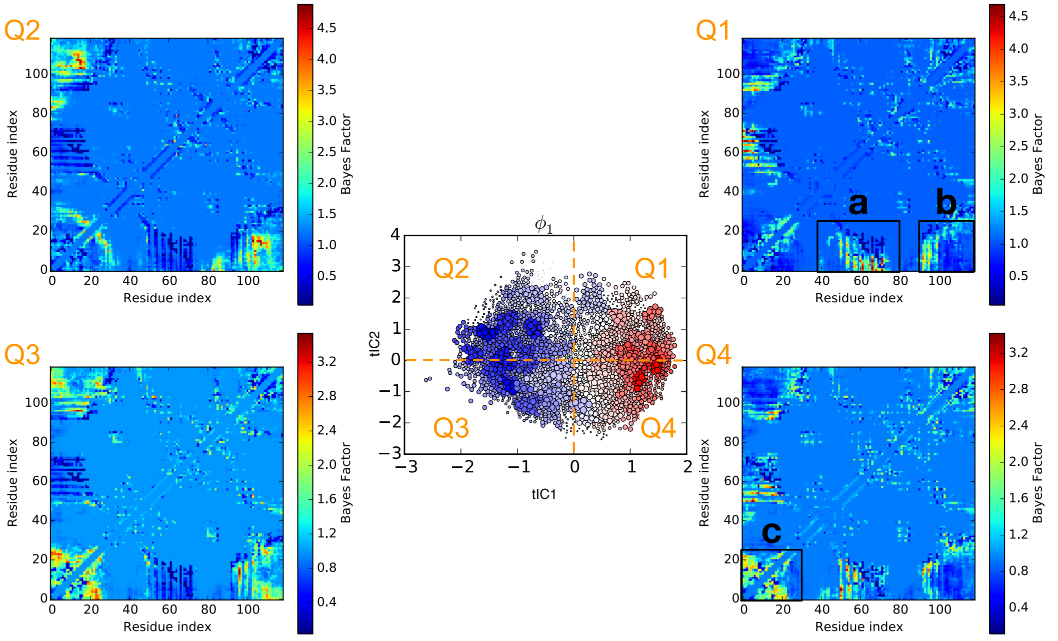

Figure S6. Bayes Factors of inter-residue contacts calculated for each quadrant in (tIC_1, tIC_2)-space. At center is shown the 2000 MSM microstates projected to the tICA landscape, divided into quadrants Q1, Q2, Q3 and Q4. (a) Differences in the Bayes factors for Q2+Q3 versus Q1+Q4 reveal that contacts are formed between the N-terminus and the active site helix α_2 along tIC_1, the slowest relaxation mode, and (b) contacts are lost between the N- and C-terminus along tIC_1. Differences in the Bayes factors for Q1+Q2 versus Q3+Q4 reveal that (c) conformational changes along tIC_2 correspond to structuring in the N-terminus.

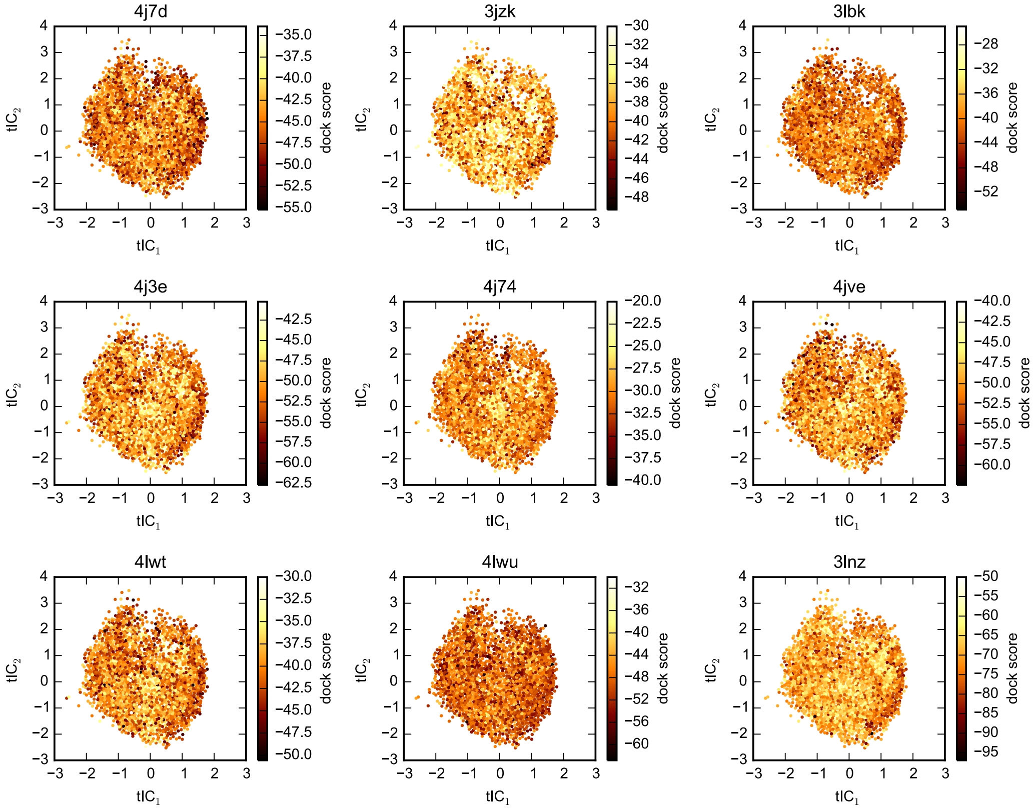

Figure S7. DOCK scores projected onto the 2D tICA landscape for all ligands. Results for p53 (1ycr) are shown in the main text.

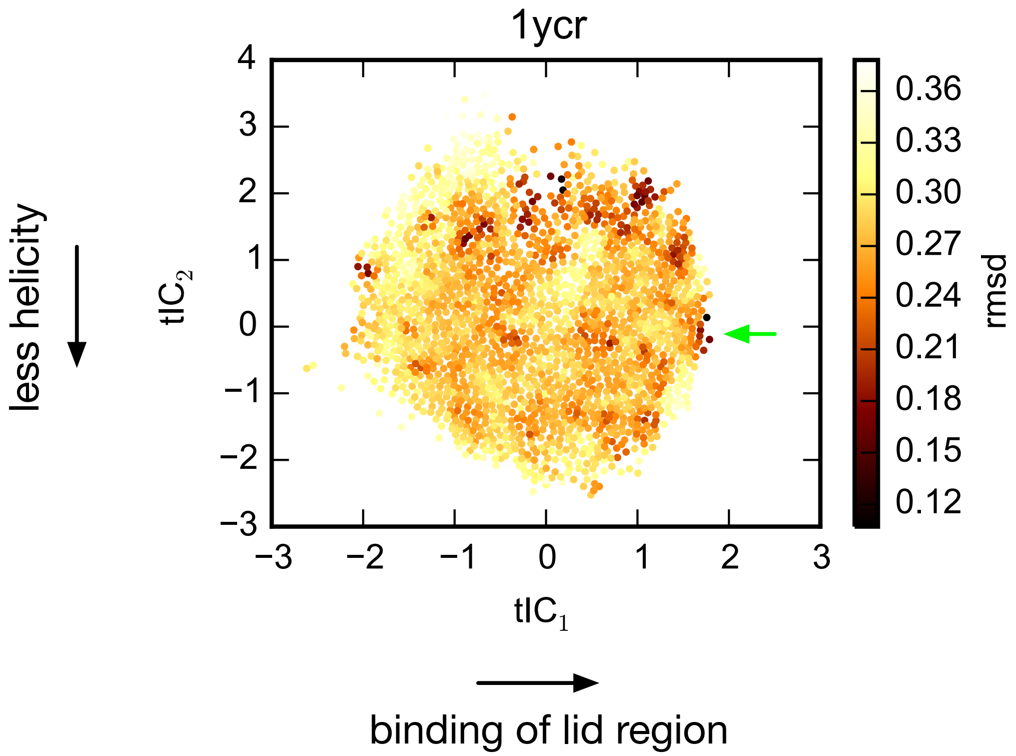

Figure S8. Backbone RMSDs of MDM2 lid residues 11-17 (DGAVTTS) to the bound-state p53 helix, shown for all 2000 MSM microstates on the tICA landscape. In the unbound state, this sequence has low propensity to form a p53-like helix, with two exceptions: (1) There is some residual helicity in the unbound state, which is lost along the ϕ_2 eigenmode relaxation (positive to negative tIC$_2$ values, see Figure S4) as the N-terminus self-associates, and (2) association of the lid region with the p53 binding cleft induces structuring of residues 11-17 to a conformation very similar to bound p53 (green arrow).
